# Grey matter atrophy in patients with benign multiple sclerosis

**DOI:** 10.1002/brb3.2679

**Published:** 2022-06-28

**Authors:** Marja Niiranen, Juha Koikkalainen, Jyrki Lötjönen, Tuomas Selander, Antti Cajanus, Päivi Hartikainen, Sakari Simula, Ritva Vanninen, Anne M. Remes

**Affiliations:** ^1^ Neuro Center, Neurology Kuopio University Hospital Kuopio Finland; ^2^ Combinostics Ltd Tampere Finland; ^3^ Institute of Clinical Medicine – Neurology University of Eastern Finland Kuopio Finland; ^4^ Science Service Center Kuopio University Hospital Kuopio Finland; ^5^ Department of Neurology Mikkeli Central Hospital Mikkeli Finland; ^6^ Institute of Clinical Medicine – Radiology University of Eastern Finland Kuopio Finland; ^7^ Department of Radiology Imaging Center, Kuopio University Hospital Kuopio Finland; ^8^ Unit of Clinical Neuroscience, Neurology University of Oulu Oulu Finland; ^9^ Medical Research Center Oulu University Hospital Oulu Finland

**Keywords:** benign multiple sclerosis, brain atrophy, corpus callosum index, thalamus, MRI

## Abstract

**Background:**

Brain atrophy appears during the progression of multiple sclerosis (MS) and is associated with the disability caused by the disease.

**Methods:**

We investigated global and regional grey matter (GM) and white matter (WM) volumes, WM lesion load, and corpus callosum index (CCI), in benign relapsing‐remitting MS (BRRMS, *n* = 35) with and without any treatment and compared those to aggressive relapsing‐remitting MS (ARRMS, *n* = 46). Structures were analyzed by using an automated MRI quantification tool (cNeuro®).

**Results:**

The total brain and cerebral WM volumes were larger in BRRMS than in ARRMS (*p* = .014, *p* = .017 respectively). In BRRMS, total brain volumes, regional GM volumes, and CCI were found similar whether or not disease‐modifying treatment (DMT) was used. The total (*p* = .033), as well as subcortical (*p* = .046) and deep WM (*p* = .041) lesion load volumes were larger in BRRMS patients without DMT. Cortical GM volumes did not differ between BRRMS and ARRMS, but the volumes of total brain tissue (*p* = .014) and thalami (*p* = .003) were larger in patients with BRRMS compared to ARRMS. A positive correlation was found between CCI and whole‐brain volume in both BRRMS (*r* = .73, *p* < .001) and ARRMS (*r *= .80, *p* < .01).

**Conclusions:**

Thalamic volume is the most prominent measure to differentiate BRRMS and ARRMS. Validation of automated quantification of CCI provides an additional applicable MRI biomarker to detect brain atrophy in MS.

## Graphical abstract

We investigated global and regional grey matter (GM) and white matter (WM) volumes, WM lesion load, and corpus callosum index (CCI), in benign relapsing.]remitting MS (BRRMS, *n* = 35) with and without any treatment and compared those to aggressive relapsing.]remitting MS (ARRMS, *n* = 46). Structures were analyzed by using an automated MRI quantification tool (cNeuro®). In our study thalamic volume was the most prominent measure to differentiate BRRMS and ARRMS. Validation of automated quantification of CCI provides an additional applicable MRI biomarker to detect brain atrophy in MS.

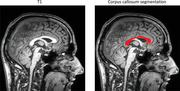

## 1 INTRODUCTION

Multiple sclerosis (MS) is a chronic inflammatory and neurodegenerative disease of the central nervous system (CNS), leading to diverse clinical outcomes and disability. Focal inflammatory white matter (WM) lesions due to demyelination are the radiological evidence of the disease activity, are the basis of the diagnosis, and often correlate with the clinical symptoms (McDonald et al., 2001; Polman et al., 2011; Poser et al., 1983). Current disease‐modifying treatments (DMT) target the inflammatory component of MS pathology. Early and optimal treatment is crucial to control the inflammatory phase of the disease and control the severity of the neurodegenerative phase (Coles et al., 2006; Freedman, 2008; Lassmann et al., 2012). Treatments with fingolimod and alemtuzumab have been shown to reduce the progression of brain atrophy (Coles et al., 2017; De Stefano et al., 2016; Gaetano et al., 2018; Yousuf et al., 2017), while most other DMTs have shown only minimal or controversial results. MRI examinations have traditionally focused on detecting and characterizing the WM lesions to follow‐up disease activity and treatment outcome.

The whole‐brain and regional grey matter (GM) atrophy measurements have recently become an essential part of the imaging domain in MS, indicating that the degenerative neuroaxonal component plays a significant role in the irreversible physical and cognitive disability in MS (Bjartmar et al., 2003). Therefore, the reduction of the rate of brain atrophy is also an essential target of MS treatments to minimize the permanent disability. The volume loss of GM is a result of the slowly ongoing neurodegenerative process. Brain atrophy occurs in normal aging at the rate of 0.1–0.3% per year, but in MS this annual rate is higher compared to age‐related measures: 0.5–1.3% at all stages of the disease (De Stefano et al., 2014; Giorgio et al., 2010). Pronounced GM atrophy can be seen already at the early stages of the relapse‐onset MS (Bergsland et al., 2012; Calabrese et al., 2007) and primary‐progressive MS (PPMS) (Sastre‐Garriga et al., 2004).

In addition to global GM atrophy, regional deep GM atrophy has been associated with the evolution of definite MS and disability progression in early relapsing‐remitting MS (RRMS), and with the evolution of PPMS (Mesaros et al., 2011; Zivadinov et al., 2013). Especially thalamic atrophy seems to appear early in MS and is associated with cognitive decline (Houtchens et al., 2007; Schoonheim et al., 2015). Thalamus is a vital relay nucleus with cortical and subcortical connections and, thus, a critical location in MS. MRI studies have strengthened the previous histopathologic findings of axonal disconnection in major thalamic tracts and thalamic demyelinating lesions (Cifelli et al., 2002; Harrison et al., 2015). Thalamic volume decline has been reported to be present consistently across MS subtypes and throughout the disease, correlating with whole‐brain atrophy (Azevedo et al., 2018).

Corpus callosum (CC) contains millions of axons, which are mainly myelinated. It is significantly affected by focal demyelination and Wallerian degeneration in the pathogenesis of MS (Evangelou et al., 2000). Corpus callosum atrophy is associated with the level of disability in MS and correlates with other measures of brain atrophy and GM atrophy (Klawiter et al., 2015; Vaneckova et al., 2012; Yaldizli et al., 2010). At the same time, CC is resistant to age‐related changes in healthy individuals (Pozzilli et al., 1994; Sullivan et al., 2001). As a sharply demarcated WM structure, CC can be delicately identified in conventional MRI. Corpus callosum index (CCI) and corpus callosum area (CCA) appear to be reliable methods for the assessment of CC atrophy in MRI (Granberg et al., 2015; Klawiter et al., 2015; Yaldizli et al., 2010).

Although different visual rating scales are used in clinical work to quantify brain atrophy, they are relatively coarse and subject to inter‐rater variability. Automated quantification tools providing brain and lesion load volumetry may help to evaluate the prognosis and activity of the MS and monitor the drug responses.

The clinical course of MS is variable. A proportion of MS patients show minimal disability even decades after the onset of MS symptoms, and this entity of the so‐called benign MS has been debated since the 1950s (Ramsaransing & De Keyser, 2006). There are no clinical prognostic markers to predict the benign course of MS. Controversially, a proportion of patients with clinical benign MS phenotype have a large WM T2 lesion load (Strasser‐Fuchs et al., 2008). More significant brain volume loss has also been reported in benign MS patients compared to healthy subjects (Rovaris et al., 2008). The reduction of brain volume in benign MS disease has been even comparable to secondary‐progressive MS (SPMS) as the course of the disease has been long (Rovaris et al., 2008). Reduction of thalamic volume (Rovaris et al., 2009) and GM volumes in subcortical and frontoparietal regions (Mesaros et al., 2008) in benign MS compared to healthy controls has been reported, but no studies are validating the CC as an atrophy marker in benign MS. The effect of DMT on brain atrophy in benign MS has not been studied.

In this study, we used an automated MRI quantification tool (cNeuro®) to evaluate global and regional GM volumes and WM lesion load in benign MS. Also, the CCI was evaluated as a marker of brain atrophy.

## 2 MATERIALS AND METHODS

### 2.1 Patients

**FIGURE 1:**
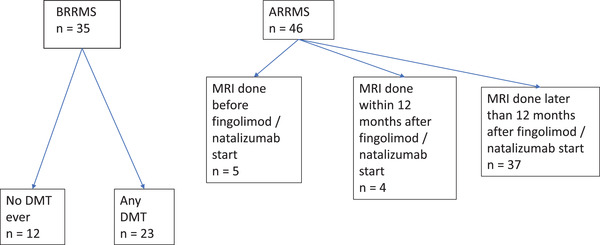
Patients of the study

The study population consists of patients with benign relapsing‐remitting MS (BRRMS, *n* = 35) and aggressive relapsing‐remitting MS (ARRMS, *n* = 46) from Neurology Outpatient Clinics of Kuopio University Hospital and Mikkeli Central Hospital (Figure 1). The regional ethics review board in Kuopio University Hospital, Finland, approved this study (decision 44/2014). Written informed consents were not gathered as all study data was obtained from the clinical patient records. All patients were diagnosed to have clinically definite MS, according to Poser (Poser et al., 1983) or McDonald criteria (McDonald et al., 2001; Polman et al., 2011).

A patient was classified to have BRRMS when the Expanded Disability Status Scale (EDSS) score was ≤ 3 and disease duration ≥ 10 years, a commonly used definition for benign MS (Glad et al., 2010). These patients had never used any DMT or were stable with the first‐line DMT (dimethyl fumarate, glatiramer acetate, interferon, or teriflunomide). A patient was classified to have ARRMS if the patient had a highly active clinical course of MS (several or very disabling relapses in early disease history and MRI activity) and had been treated with fingolimod or natalizumab.

Demographic details and MS disease history were retrospectively collected from the patient records (Table 1). Clinical evaluation, including EDSS, was performed by an experienced neurologist at the time of MRI scanning (Kurtzke, 1983). Disease duration was defined as the time elapsed from the onset symptoms of MS until the MRI scanning. MRI and clinical data were collected from years 2007 to 2017.

**TABLE 1:** Baseline demographics of the patients

	Benign MS	Aggressive MS	
Variable^a^	*n* = 35	*n* = 46^b^	*p*
Gender, female, *n* (%)	28 (80)	33 (71.7)	.393
Age at onset symptoms, years (range)	32.8 (14–51)	30.5 (14–59)	.248
Age at time of MRI, years (range)	51.0 (32–70)	43.2 (21–69)	<.001
Duration of disease at the time of MRI years (range)	18.2 (12–33)	12.6 (0–36)	<.001
Number of relapses at the time of MRI (median; range)	4.0 (1–10)	5.0 (1–43)	.004
EDSS score at the time of MRI (median; range)	2.0 (0–3.0)	2.8 (0–8.5)	<.001
Onset symptoms of MS, *n* (%)^c^			
Optic neuritis	8 (22.9)	14 (30.4)	.448
Sensory symptoms	10 (28.6)	7 (15.2)	.144
Motor paresis	5 (14.3)	11 (23.9)	.281
Cerebellar/brainstem symptoms	9 (25.7)	15 (32.6)	.501
Myelitis	11 (31.4)	7 (15.2)	0.082
DMT at the time of MRI scanning, *n* (%)			
None	13 (37.1)	9 (19.6)	
Interferon or glatiramer acetate	19 (54.3)	5 (10.9)	
Teriflunomide or dimethyl fumarate	3 (8.6)	3 (6.5)	
Natalizumab or fingolimod	0	25 (54.3)	
Alemtuzumab	0	4 (8.7)	

Abbreviations: DMT, disease‐modifying treatment; EDSS, Expanded Disability Status Scale; MRI, magnetic resonance imaging; MS, multiple sclerosis.

^a^ Values are mean and range unless other indicated.

^b^ In medication history only fingolimod *n* = 14, only natalizumab *n* = 15, both fingolimod and natalizumab *n* = 17.

^c^ Total exceeds 100% since polysymptomatic relapses occurred.

All patients were clinically stable within 1 month before MRI scanning (neither clinical relapses nor cortisone treatments).

### 2.2 MRI acquisition and analysis

The subjects were referred to MRI with clinical indications. The time point of the imaging varied due to the retrospective nature of the study. Several different MRI scanner models (1.5‐ or 3‐Tesla) were used. Scanner models were evenly distributed in both ARRMS and BRRMS. In BRRMS, 20% of examinations were performed with 3T scanners, 43.5% in ARRMS. The imaging protocol included a three‐dimensional T1‐weighted gradient‐echo sequence (3D T1‐w) and a fast fluid‐attenuated inversion recovery (FLAIR) sequence. The voxel size varied between 0.4−1.6 × 0.4−1.6 × 0.5−2.2 mm in T1 images and 0.4−1.3 × 0.4−1.3 × 0.6−7.0 mm in FLAIR images. Altogether 41% of the 3D T1‐w images appropriate for volumetric analysis were scanned with gadolinium (Gd) enhancement. Gd images were evenly distributed in ARRMS and BRRMS groups. The latest MRI examination, including 3D T1‐w images, was chosen to obtain the longest period possible counted from the onset symptoms.

**FIGURE 2:**
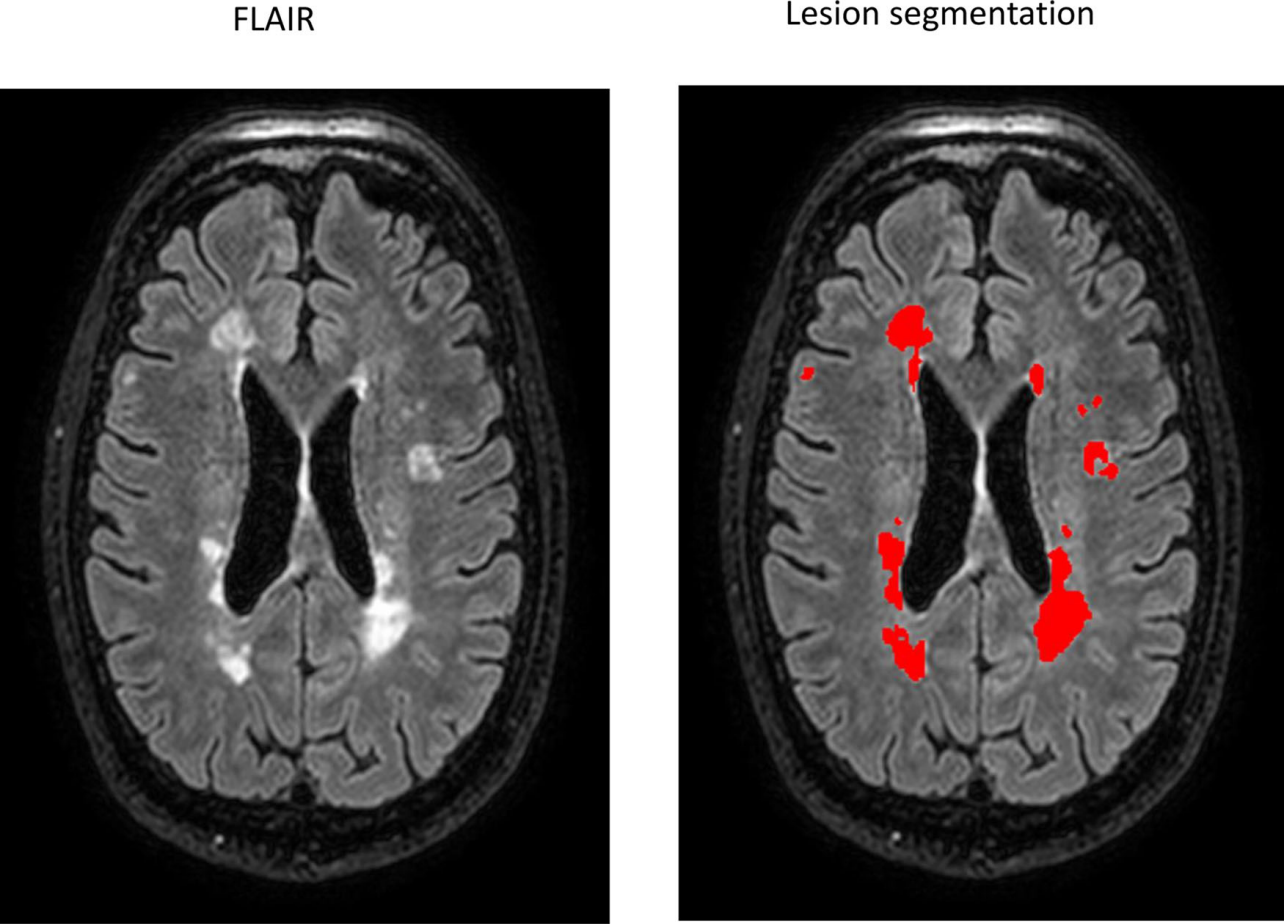
Lesion segmentation in the cNeuro® quantification tool

**FIGURE 3:**
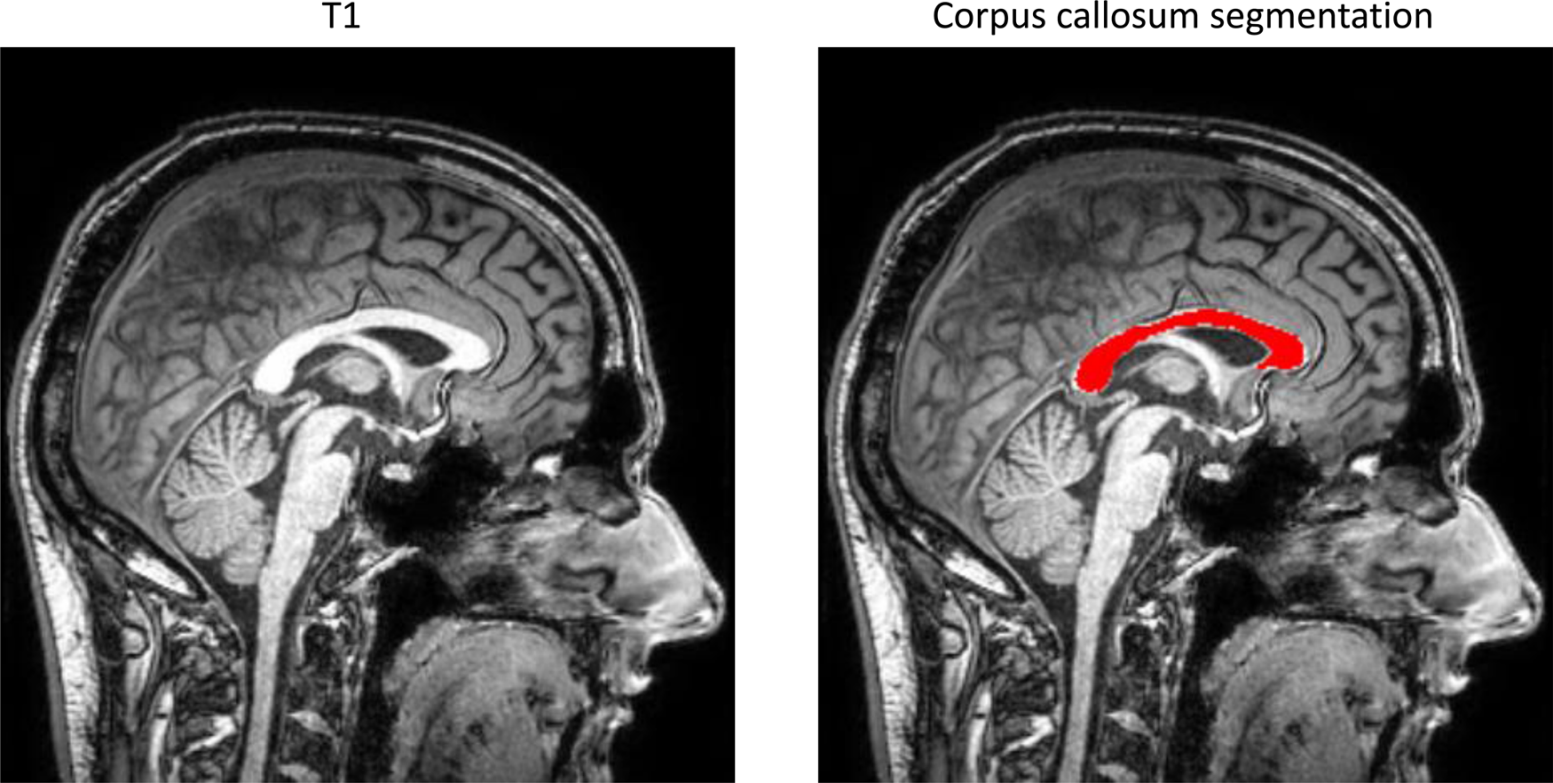
Corpus callosum segmentation in the cNeuro® quantification tool

A set of 328 different volumetry and voxel‐based morphometry imaging biomarkers was extracted from T1‐weighted and FLAIR images using the cNeuro® MRI quantification tool (Combinostics Oy, Tampere, Finland) (Lotjonen et al., 2010). Images were segmented into 133 brain regions using the multiatlas segmentation method (102 cortical and 31 subcortical regions) (Hanninen et al., 2019; Koikkalainen et al., 2016; Lotjonen et al., 2010). In this study, results for 27 imaging biomarkers are reported. The WM lesions were segmented as previously described (Koikkalainen et al., 2016; Wang et al., 2012), and the lesion volume is reported globally and regionally for the following brain regions: periventricular, subcortical, deep white matter, pons, and cerebellum (Figure 2). The method uses the state‐of‐the‐art lesion filling technique which removes lesions from images before T1 segmentation. All the quantified variables were normalized regarding age, gender, and head size (Buckner et al., 2004; Cole & Green, 1992). The extraction of the CCI was not available in cNeuro. For the automated computation of the CCI (Goncalves et al., 2018; Yaldizli et al., 2010), six landmarks were first manually located on a mean anatomical template. The T1 image was first affinely and then nonrigidly registered with the mean anatomical template, and the landmarks were propagated accordingly to the T1 image for the automated computation of individual CCI (Figure 3).

### 2.3 Statistical analysis

Statistical analyses were performed with IBM SPSS Statistics for Windows version 24 (IBM Corp, Armonk, NY). Baseline demographics were expressed as means with ranges or frequencies with percentages. Differences between groups were tested with the Mann‐Whitney U test and the chi‐square test. Brain MRI segmentation volumetric results between groups were compared by analysis of covariance (ANCOVA) model. In the ANCOVA model, age, the length of disease duration, and Gd‐enhancement (with or without Gd) functioned as adjusting variables. Means and standard deviations were reported. In addition, the regression coefficients with *p*‐values and standardized Betas were expressed to measure effect size difference between study groups. *p*‐Values < .05 indicated statistically significant results.

## 3 RESULTS

### 3.1 Clinical characteristics

Patients in the BRRMS group were older (mean age 51.0 years, range 32–70) than in the ARRMS group (mean 43.2 years, range 21–69) at the time of MRI (*p* < .001), the duration of the disease was longer (18.2 years vs. 12.6 years, respectively, *p* < .001), and they had had fewer relapses (median 4.0 vs. 5.0, *p* = .004). Onset symptoms did not differ between BRRMS and ARRMS groups (Table 1).

Patients with BRRMS had used none or only first‐line DMTs through all their medical history. Altogether 12 patients out of 35 in the BRRMS group (34.3%) had not been treated with any DMT from the time of onset symptoms until the time of MRI scanning (Figure 1). The mean age of patients in BRRMS without any treatments was 54.6 years (range 46–66) and 49.1 years (range 32–70) in BRRMS with some history of DMT (*p* = .120). Disease duration was slightly longer in this nontreated subgroup (mean 20.7 years vs. 16.9 years, *p* = .027). The median number of relapses through the disease history was 3.0 (range 1–5) in patients who were without any DMT compared to 4.0 (range 2–10) in patients with any DMT (*p* = .050). EDSS levels did not differ between the BRRMS groups (median 1.75 vs. 2.00, respectively, *p* = .861).

The majority of patients with ARRMS were using fingolimod or natalizumab (*n* = 25, 54.3%) at the time of MRI examination (Table 1). Brain imaging was done after the initiation of fingolimod or natalizumab in 41 (89.1%) patients with ARRMS (Figure 1). The time of MRI examination in relation to the initiation of highly effective DMT varied due to the retrospective nature of the study, and there was variation in the MRI imaging protocols: in five patients, there were applicable MRI scans with 3D T1‐w images only from the time before the start of fingolimod or natalizumab (Figure 1).

### 3.2 Whole‐brain volume, GM and WM volumes and regional GM volumes in BRRMS and ARRMS

Total brain tissue volume was larger in patients with BRRMS (mean 1098.42 ml, SD 52.82) compared to ARRMS (mean 1069.4 ml, SD 60.09), (*p* = .014). Both the cerebral (mean 369.82 ml, SD 37.76) (*p* = .017) and cerebellar (mean 22.12 ml, SD 3.58) (*p* = .015) WM volumes were larger in patients with BRRMS, while cortical GM volumes did not differ between the groups. Thalamic volume was larger in BRRMS (mean 12.94 ml, SD 1.9) than in the ARRMS group (mean 11.82 ml, SD1.82) (*p* = .003). Total and regional volumes are given in Table 2.

**TABLE 2:** MRI volumetry in patients with BRRMS and ARRMS

Variable	BRRMS	ARRMS	B	*p*	Beta
*n*	35	46			
Volumes, ml (mean; SD)					
Brain tissue (total)	1098.42 (52.82)	1069.4 (60.09)	−31.01	.014	−.26
Brain WM (total)	369.82 (37.76)	351.42 (36.46)	−21.92	.017	−.29
Cortical GM (total)	493.8 (33.47)	489.14 (47.33)	−2.15	.778	−.03
Cerebral GM	522.05 (35.99)	515.26 (49.30)	−4.29	.598	−.05
Cerebellar GM	97.12 (9.54)	93.94 (7.76)	−4.32	.039	−.25
Cerebellar WM	22.12 (3.58)	20.68 (2.74)	−1.9	.015	−.30
Cerebrospinal fluid (total)	57.87 (26.55)	63.02 (19.66)	5.63	.306	.12
Lobar volumes, ml (mean; SD)					
Frontal lobes	191.54 (15.48)	193 (18.64)	2.26	.500	.07
Temporal lobes	121.08 (7.14)	118.5 (10.9)	−2.14	.294	−.11
Parietal lobes	107.74 (8.2)	104.2 (11.98)	−2.48	.252	−.12
Occipital lobes	72.74 (7.36)	72.4 (9.94)	−0.16	.927	−.01
Regional volumes, ml (mean; SD)					
Postcentral gyrus	17.5 (1.86)	16.94 (2.48)	−0.32	.481	−.07
Postcentral gyrus (medial segment)	1.2 (0.3)	1.18 (0.28)	0.08	.248	.13
Precentral gyrus	22.5 (3.04)	23.04 (2.86)	0.5	.426	.08
Precentral gyrus (medial segment)	4.58 (0.64)	4.7 (0.78)	0.28	.084	.19
Medial temporal lobes	18.84 (1.88)	18.74 (2.02)	−0.4	.411	−.10
Hippocampus	6.5 (0.92)	6.4 (0.86)	−0.2	.369	−.11
Thalamus	12.94 (1.9)	11.82 (1.82)	−1.26	.003	−.33
Anterior cingulate gyrus	8.42 (1.38)	8.74 (1.68)	0.44	.226	.14
Posterior cingulate gyrus	9.56 (1.18)	9.24 (1.2)	−0.38	.170	−.16
CCI	0.34 (0.04)	0.32 (0.05)	−0.03	.011	−.29
Volumes of WM lesions, ml (mean; SD)					
Total	14.1 (10.73)	20.01 (11.23)	6.28	.020	.28
Periventricular	2.88 (3.6)	5.83 (5.37)	3.94	.001	.40
Subcortical	0.24 (0.49)	0.28 (0.46)	0.03	.791	.03
Deep white matter	8.39 (7.01)	10.67 (6.38)	1.99	.214	.15
Pons	0 (0.01)	0 (0.01)	0	.207	.15
Cerebellar	0 (0.01)	0 (0)	0	.923	.01

*Note*: B, coefficient B in regression analysis for group difference. Difference from ARRMS to BRRMS adjusted with duration of disease and Gadolinium‐enhancement

Abbreviations: ARRMS, aggressive MS; Beta, standardized coefficient between groups; BRRMS, benign MS; CCI, corpus callosum index; GM, grey matter; MRI, magnetic resonance imaging; *p*, *p*‐value for group difference, adjusted with age, time from onset symptoms, and Gadolinium‐enhancement; WM, white matter.

### 3.3 Volumes of WM lesions and CCI in BRRMS and ARRMS

**FIGURE 4:**
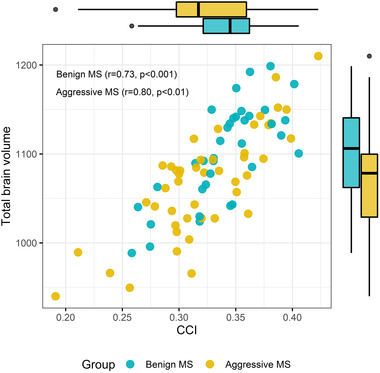
Correlation between corpus callosum index (CCI) and total brain volume

**FIGURE 5:**
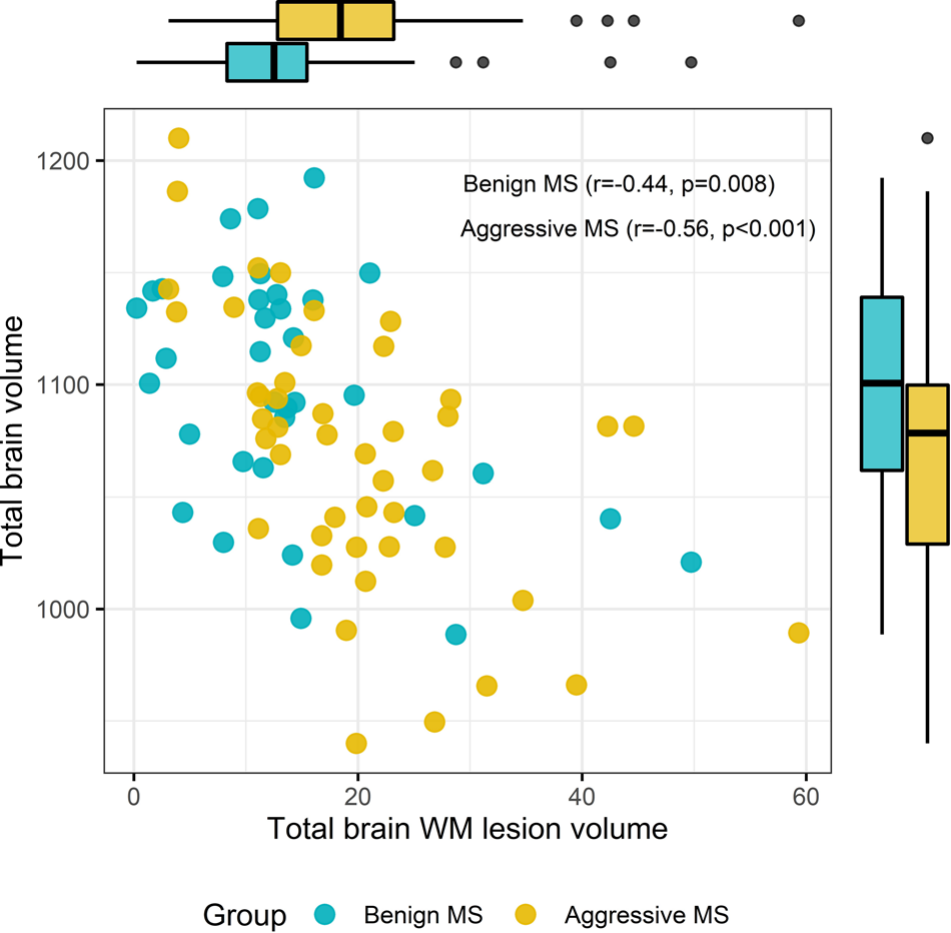
Correlation between total white matter (WM) lesion volume and total brain volume

The total volume of WM lesion load was larger in ARRMS (mean 20.01 ml, SD 11.23) compared to BRRMS (mean 14.1 ml, SD 10.73) (*p* = .020). Also, periventricular WM lesion volume in ARRMS (mean 5.83 ml, SD 5.37) was larger than in BRRMS (mean 2.88 ml, SD 3.6) (*p* = .001). CCI was higher in BRRMS (mean 0.34, SD 0.04) than in ARRMS (mean 0.32, SD 0.05), (*p* = .011) (Table 2). There was a positive correlation between CCI and whole‐brain volume in both BRRMS (*r* = .73, *p* < .001) and ARRMS (*r* = .80, *p* < .01) (Figure 4). There was a negative correlation between total brain tissue volume and WM lesion volume in both BRRMS (*r* = −.44, *p* = .008) and ARRMS (*r* = −.56, *p* < .001). There was no difference between these correlations (*p* = .516) (Figure 5).

### 3.4 DMT use, brain volumes and WM lesion volumes in BRRMS

There were no differences between the treated and nontreated BRRMS patients in total brain volumes, neither in regional GM volumes. Also, CCI did not differ between these subgroups. The total WM lesion volumes (*p* = .033), as well as regional WM lesion volumes in the subcortical area (*p* = .046) and deep white matter (*p* = .041), were larger in the subgroup of patients without DMT use (Table 3).

**TABLE 3:** MRI volumetry of patients with BRRMS

Variable, *n*	With DMT	Without DMT	B	*p*	Beta
	23	12			
Volumes, ml (mean; SD)					
Brain tissue (total)	1110.05 (50.1)	1076.12 (52.73)	21.43	.241	.20
Brain WM (total)	377.4 (35.3)	357.2 (40.64)	22.84	.146	.29
Cortical GM (total)	500.61 (35.24)	480.77 (26.38)	4.31	.600	.06
Cerebral GM	529.54 (37.29)	507.7 (29.69)	6.66	.483	.09
Cerebellar GM	97.9 (9.8)	95.66 (9.28)	1.26	.754	.06
Cerebellar WM	22.66 (2.9)	21.08 (4.58)	1.06	.451	.14
Cerebrospinal fluid (total)	52.77(21.62)	67.66 (32.93)	−26.62	.215	−.24
Lobar volumes, ml (mean; SD)					
Frontal lobes	194.28 (16.52)	186.3 (12.28)	3.30	.434	.10
Temporal lobes	122.22 (6.48)	118.9 (8.12)	0.06	.978	.00
Parietal lobes	109.02 (8.52)	104.96 (7.1)	1.60	.573	.09
Occipital lobes	74.08 (8.04)	70.16 (5.18)	0.12	.960	−.01
Regional volumes, ml (mean; SD)					
Postcentral gyrus	17.68 (2.1)	17.18 (1.34)	−0.28	.655	.00
Postcentral gyrus (medial segment)	1.18 (0.34)	1.22 (0.22)	0.02	.922	−.02
Precentral gyrus	22.84 (3.38)	21.88 (2.26)	0.04	.968	−.01
Precentral gyrus (medial segment)	4.56 (0.72)	4.62 (0.5)	−0.16	.506	−.12
Medial temporal lobes	19.28 (2.0)	18.00 (1.36)	1.28	.097	.33
Hippocampus	6.68 (0.98)	6.18 (0.7)	0.54	.159	.28
Thalamus	13.28 (1.52)	12.32 (2.42)	0.60	.414	.15
Anterior cingulate gyrus	8.6 (1.38)	8.06 (1.4)	0.50	.379	.17
Posterior cingulate gyrus	9.7 (1.18)	9.26 (1.16)	0.18	.682	.07
CCI	0.35 (0.03)	0.32 (0.04)	0.02	.187	.25
Volumes of WM lesions, ml (mean; SD)					
Total	11.31 (6.09)	19.46 (15.29)	−9.12	.033	−.41
Periventricular	1.9 (2.47)	4.76 (4.69)	−2.74	.059	−.41
Subcortical	0.11 (0.17)	0.49 (0.76)	−0.39	.046	−.38
Deep white matter	6.76 (3.96)	11.52 (10.21)	−5.66	.041	−.39
Pons	0 (0)	0.01 (0.01)	−0.01	0.050	−.37
Cerebellar	0 (0.01)	0 (0)	0.00	.580	.11

*Note*: B = coefficient B in regression analysis for group difference. Difference between BRRMS without DMT and BRRMS with DMT adjusted with duration of disease and Gd‐enhancement.

Abbreviations: Beta, standardized coefficient between groups; CCI, corpus callosum index; GM, grey matter; MRI, magnetic resonance imaging; *p*, adjusted *p*‐value for group difference; WM, white matter.

### 3.5 Whole‐brain and regional volumes, WM lesion volumes and CCI in subgroups of ARRMS compared to BRRMS

Because the time of MRI in relation to the initiation of highly effective DMT varied within the ARRMS group, we did a subgroup analysis between BRRMS and the three different subgroups of ARRMS given in Figure 1. These results in volumetry are given as supplementary data (Table 4). Smaller thalamic volumes and periventricular WM lesion volumes compared to BRRMS were detected in the subgroups of ARRMS scanned before and those scanned for more than 12 months after the initiation of highly effective DMT.

**TABLE 4:** Supplementary data of MRI volumetry, in patients across BRRMS and three subgroups of ARRMS

Variable	BRRMS, all	ARRMS, MRI before initiation of DMT	B	*p*	ARRMS, MRI within 12 months after initiation of DMT	B	*p*	ARRMS, MRI more than 12 months after initiation of DMT	B	*p*
*n*	35	5			4			37		
Volumes, ml (mean; SD)										
Brain tissue (total)	1098.42 (52.82)	1085.94 (31.49)	31.49	.099	1101.46 (66.6)	−24.2	1.000	1063.70 (62.04)	−30	.057
Brain white matter (total)	370.86 (37.62)	351.0 (36.46)	−28.8	.423	354.42 (48.44)	−22.8	.789	352.42 (35.94)	−23	.045
Cortical grey matter (total)	493.8 (33.47)	510.73 (23.4)	−18.1	.786	529.23 (32.24)	15.14	1.000	481.89 (48.53)	−2	1.000
Cerebral grey matter	522.05 (35.99)	536.77 (25.12)	−22	.606	558.08 (35.03)	14.69	1.000	507.72 (50.39)	−4	1.000
Cerebellar grey matter	97.12 (9.54)	89.24 (5.38)	−12.3	.018	92.56 (5.28)	7.32	.330	94.72 (8.12)	−2	.759
Cerebellar white matter	22.12 (3.58)	20.28 (2.9)	−3.42	.111	20.2 (1.48)	−2.64	.363	20.76 (2.88)	1.6	.123
Cerebrospinal fluid (total)	57.87 (26.55)	67.84 (23.65)	21.24	.201	44.89 (14.84)	−6.13	1.000	64.33 (19)	5.3	1.000
Lobar volumes, ml (mean; SD)										
Frontal lobes	191.54 (15.48)	199.98 (10.04)	−6.14	1.000	203.82 (17.5)	3.74	1.000	190.9 (19.32)	3.1	1.000
Temporal lobes	121.08 (7.14)	119.56 (5.88)	−7.68	.231	126.64 (9.3)	2.02	1.000	117.48 (11.3)	−2	1.000
Parietal lobes	107.74 (8.2)	110.76 (5.92)	−3.28	1.000	114.78 (9.52)	3.48	1.000	102.18 (12.06)	−3	.429
Occipital lobes	72.74 (7.36)	72.58 (6.08)	−0.44	1.000	80.54 (7.54)	4.16	.867	70.68 (9.96)	−0	1.000
Regional volumes, ml (mean; SD)										
Postcentral gyrus	17.5 (1.86)	17.8 (1.22)	−1.14	.720	18.54 (2.16)	0.22	1.000	16.66 (2.56)	−0	1.000
Postcentral gyrus (medial segment)	1.2 (0.3)	1.12 (0.3)	0.06	1.000	1.26 (0.2)	0.1	1.000	1.18 (0.3)	0.1	1.000
Precentral gyrus	22.5 (3.04)	23.54 (3.26)	−1.5	.753	22.84 (3.74)	−1.18	1.000	22.98 (2.8)	0.9	.480
Precentral gyrus (medial segment)	4.58 (0.64)	4.88 (0.78)	0.02	1.000	4.6 (0.62)	−0.12	1.000	4.7 (0.8)	0.3	.150
Medial temporal lobes	18.84 (1.88)	18.1 (2.0)	−0.74	1.000	19.12 (3.2)	0.22	1.000	18.78 (1.94)	−0	1.000
Hippocampus	6.5 (0.92)	6.52 (0.88)	−0.06	1.000	6.48 (0.94)	−0.08	1.000	6.38 (0.88)	−0	1.000
Thalamus	12.94 (1.9)	12.04 (1.46)	−2.24	.048	12.58 (1.56)	−1.22	.615	11.7 (1.9)	−1	.006
Anterior cingulate gyrus	8.42 (1.38)	10.24 (2.16)	1.42	.204	9.68 (1.12)	2.6	.594	8.44 (1.56)	0.2	1.000
Posterior cingulate gyrus	9.56 (1.18)	10.12 (0.9)	0.012	1.000	9.28 (1.26)	−0.54	1.000	9.12 (1.22)	−0	.684
CCI	0.34 (0.04)	0.33 (0.03)	−0.04	.138	0.37 (0.04)	0.01	1.000	0.32 (0.05)	−0	.015
Volumes of WM lesions, ml (mean; SD)										
Total	14.1 (10.73)	23.2 (13.07)	14.72	.033	16.14 (13.22)	5.58	1.000	19.99 (11)	6.1	.084
Periventricular	2.88 (3.6)	6.42 (6.21)	6.08	.039	3.49 (6.14)	2.27	1.000	6.0 (5.28)	3.6	.006
Subcortical	0.24 (0.49)	0.32 (0.63)	0.23	1.000	0.02 (0.02)	−0.13	1.000	0.31 (0.46)	0.1	1.000
Deep white matter	8.39 (7.01)	13.61 (7.4)	8.04	.066	9.32 (6.68)	2.67	1.000	10.42 (6.29)	1.9	.798
Pons	0 (0.01)	0.01 (0.02)	0.01	.357	0 (0)	0	1.000	0 (0.01)	0	1.000
Cerebellar	0 (0.01)	0 (0)	0	1.000	0 (0)	0	1.000	0 (0)		1.000

*Note*: B = coefficient B in regression analysis for group difference. Difference from ARRMS to BRRMS adjusted with duration of disease and Gd‐enhancement.

Abbreviations: ARRMS, aggressive MS; BRRMS, benign MS; CCI, corpus callosum index; *p* = *p*‐value for group difference, adjusted with time from onset symptoms and Gd‐enhancement; WM, white matter.

## 4 DISCUSSION

In this study, we focused on brain atrophy measures, global and regional GM volumes and WM lesion load, in benign MS. Further, we evaluated CCI as a measure of atrophy not reported earlier in benign MS using an automated MRI quantification tool (cNeuro®).

Total brain volumes, regional GM volumes, and CCI measures were found similar between treated and nontreated BRRMS patients. Within the BRRMS group, those patients who had never been treated with DMT had larger WM lesion volumes; even they had had a slightly lower number of relapses than patients treated with DMT. Our results support the assumption that subclinical inflammatory disease activity also occurs in seemingly benign and mild MS. So, there is justification for DMT use also in the benign course of the disease, regardless of the clinical relapse rate (Montalban et al., 2018; Ziemssen et al., 2016; Zivadinov et al., 2016). However, the evidence of the DMT effect on GM atrophy, especially in benign MS, is presently scarce. A large meta‐analysis in RRMS reported lower brain atrophy at 24 months with second‐line DMT compared to first‐line DMT but did not report specifically GM atrophy (Branger et al., 2016). In a 2‐year follow‐up study, patients treated with fingolimod showed milder GM atrophy versus nontreated patients (Yousuf et al., 2017). A longitudinal study with follow‐up MRI scanning covering a long enough period would be much more sensitive to differences and give more information on the effect of DMT in brain atrophy in a benign clinical course of MS.

As the definition of benign MS is a retrospective judgment of past disease trait, the prognostic MRI markers of the disease course are worth searching. So far, there are no established predictors for long‐term outcomes. It seems that a significant proportion of patients with benign MS develop cognitive decline, overall disability, and brain atrophy after a long follow‐up period (i.e., more than 20 years), even though there are no clinical relapses and neurological signs remain mild (Correale et al., 2012; Hirst et al., 2008; Mesaros et al., 2009; Portaccio et al., 2009; Rovaris et al., 2008; Zivadinov et al., 2016). The extent of brain atrophy in benign MS compared with an age‐matched healthy control group is scarcely investigated, reporting reduced subcortical and cortical GM in benign MS patients compared to healthy controls (Mesaros et al., 2008; Rovaris et al., 2008). Reduction of thalamic volume in benign MS compared to healthy subjects has been reported, but it may be a typical characteristic of MS itself, purely reflecting the vulnerability of the thalamus to specific damage in MS pathology (Rovaris et al., 2009). Interestingly, we found that thalamic volume was larger in BRRMS than in ARRMS, contrary to previous findings in a smaller patient study (Ceccarelli et al., 2008).

We used a set of volumetric biomarkers that were extracted from routine MRI examinations. Earlier studies have demonstrated that GM atrophy progresses despite clinically highly effective DMT, such as natalizumab (Koskimaki et al., 2018). Our study setting was planned to compare two clinically different phenotypes of MS, BRRMS, and ARMMS in a cross‐sectional study, but not to evaluate the effect of highly effective DMTs on the rate of brain atrophy in a longitudinal setting. Thus, we included the five patients in the ARRMS group scanned before the fingolimod or natalizumab treatment initiation. In the subgroup analysis, these patients had similar brain volume patterns as those who had been treated with highly active DMT for at least 1 year at an MRI time point.

To our knowledge, this is the first study using CCI as a parameter in an automated MRI quantification tool in benign MS patients. Our results are in line with the previous few reports on the negative correlation of CCI and GM atrophy (Klawiter et al., 2015) as well as CCI in benign MS (Mesaros et al., 2009). Thus, CCI seems to be an easily assessable MRI marker for brain atrophy in MS patients, and applicable in an automated tool, considering that CCI analysis done manually is time consuming and vague (Yaldizli et al., 2010). In a recent study with early relapsing MS and secondary progressive MS patients, thalamic atrophy and whole‐brain atrophy were identified as possible disease progression markers measured with the same automated MRI quantification method as used in our study (Hanninen et al., 2019).

An acceleration of volume reduction after initiation of DMTs, also referred to as pseudoatrophy, is associated mainly with natalizumab (Koskimaki et al., 2018; Miller et al., 2007). It is supposed to be a consequence of the resolution of inflammation after therapy initiation, probably due to fluid shifts (i.e., resolution of brain edema) and changes in inflammatory cells, and mostly due to white matter volume changes. However, the exact mechanism is poorly understood. The pseudoatrophy effect does not seem to occur for GM (Prinster et al., 2006). In our study, five patients in the ARRMS group had initiated fingolimod or natalizumab within 1 year before MRI scanning. These patients did not have smaller whole‐brain or WM volumes compared to the other patients within the ARRMS group. As results in CCI and thalamus volume are similar to whole‐brain volume, we assume pseudoatrophy alone does not explain the smaller whole‐brain volumes in the entire ARRMS group.

The strengths of our study include detailed clinical characteristics for each patient and thorough EDSS evaluation. The duration of the disease in patients with BRRMS clearly exceeds 10 years, which is a commonly used criterion for benign MS (Glad et al., 2010). Lack of cognitive testing may be counted as a weakness in our study. We only used EDSS as a clinical measure, which emphasizes motor functions. Part of the patients with benign clinical phenotype and minimal motor disability suffer from notable cognitive decline and depression, which should be recorded in the overall disability (Correale et al., 2012; Gonzalez‐Rosa et al., 2006; Mesaros et al., 2009).

Due to the retrospective nature of the study, the imaging protocols, scanners, and voxel sizes were variable. This might have had some impact on the imaging results, especially for the cortical GM measures rather than other volume measures. Both 1.5T and 3T imaging were analyzed, with the emphasis of 3T images in ARRMS. There is a possibility of bias due to this imbalance of scanners, but the normalization of the structures and previous studies with the same algorithm suggest that this bias has not affected the results significantly (Koikkalainen et al., 2016; Lotjonen et al., 2010). On the other hand, in a large sample size, even small differences become significant when using one single scanner and sequence with a defined single voxel size. In previous studies with FreeSurfer structural tool, the use of multiple different MRI scanners and pulse sequences did not appear to have a significant effect on cortical thickness measurements (Govindarajan et al., 2014; Potvin et al., 2017). Test–retest difference in cNeuro® MRI quantification tool between different scanners is two to three fold compared to having a one single scanner, which is equal to other methods. Also, voxel size variation does not seem to affect the results in cNeuro® MRI quantification tool. Nevertheless, we consider that our results are logical and suggest the methodology is quite robust (Kaipainen et al., 2021).

Another weakness of our study is the nature of a single‐point MRI analysis and thus, lack of longitudinal analysis. Longitudinal volumetric analysis requires that a specific MRI scanning protocol is repeated with the same scanner, which was impossible to achieve in real‐life retrospective data. Also, in this study, we did not have a healthy age‐matched control group. Almost half of the 3D T1‐w sequences were done with Gd‐enhancement, which was taken into account in the analysis. We were not able to combine information about Gd‐enhancing lesions in this volumetric study. However, we consider that this does not confound the interpretation of our results. We excluded patients who had had a clinical relapse or cortisone treatment within 1 month before MRI scanning to avoid the possible effect of evident inflammation.

## 5 CONCLUSIONS

We conclude that thalamic volume was the most prominent GM measure to differentiate BRRMS and ARRMS. Patients with BRRMS had larger whole‐brain and thalamic volumes than patients with aggressive disease course. CCI has been suggested as a marker of brain atrophy, and we conclude that an automatically quantified CCI seems to be an accessible and applicable MRI marker of brain atrophy, to be used in combination with other measures, such as whole‐brain volume and thalamic volume.

## CONFLICT OF INTEREST

JK and JL are employees and shareholders in Combinostics. JL has given educational presentations for Merck and Sanofi, paid to his institution. Other authors have no conflicts of interest.

## Data Availability

Data available on request from the authors.
